# A Sustainable Hydroxypropyl Cellulose-Nanodiamond Composite for Flexible Electronic Applications

**DOI:** 10.3390/gels8120783

**Published:** 2022-11-29

**Authors:** Elena Palmieri, Francesca Pescosolido, Luca Montaina, Rocco Carcione, Greta Petrella, Daniel Oscar Cicero, Emanuela Tamburri, Silvia Battistoni, Silvia Orlanducci

**Affiliations:** 1 Chemical Sciences Department, University of Rome “Tor Vergata”, Via della Ricerca Scientifica, 00133 Rome, Italy; 2 Consiglio Nazionale delle Ricerche—Institute of Materials for Electronics and Magnetism (CNR-IMEM), Parco Area delle Scienze 37A, 43124 Parma, Italy

**Keywords:** polymer nanocomposite, nanodiamond, flexible electronics, cellulose derivatives

## Abstract

Designing fully green materials for flexible electronics is an urgent need due to the growing awareness of an environmental crisis. With the aim of developing a sustainable, printable, and biocompatible material to be exploited in flexible electronics, the rheological, structural and charge transport properties of water-based hydroxypropyl cellulose (HPC)-detonation nanodiamond (DND) viscous dispersions are investigated. A rheological investigation disclosed that the presence of the DND affects the orientation and entanglement of cellulose chains in the aqueous medium. In line with rheological analyses, the NMR diffusion experiments pointed out that the presence of DND modifies the hydrodynamic behavior of the cellulose molecules. Despite the increased rigidity of the system, the presence of DND slightly enhances the ionic conductivity of the dispersion, suggesting a modification in the charge transport properties of the material. The electrochemical analyses, performed through Cyclic Voltammetry (CV) and Electrochemical Impedance Spectroscopy (EIS), revealed that the HPC-DND system is remarkably stable in the explored voltage range (−0.1 to +0.4 V) and characterized by a lowered bulk resistance with respect to HPC. Such features, coupled with the printability and filmability of the material, represent good requirements for the exploitation of such systems in flexible electronic applications.

## 1. Introduction

In the last decades, the growing awareness of environmental crisis triggered the scientific community to devote significant efforts to produce and design sustainable electronic devices with high performance and reduced fabrication costs [1,2,3,4,5]. In the scenario of the materials showing potential for such applications, cellulose is one of the preferred green biopolymers. Bacterial cellulose, for example, was used in electronic skin through introduction of a thermionic liquid into the hydrogel, thus modifying cellulose fibers into molecular chains, achieving a highly conductive and strong material [6]. However, for most of its application, due to its strong inter- and intramolecular hydrogen bonding, pure cellulose can be dissolved neither in water nor in many organic solvents, thus, modification and functionalization of the polymeric backbone is needed [7,8].

In particular, cellulose derivatives such as methyl cellulose (MC), carboxymethyl cellulose (CMC), hydroxy propyl cellulose (HPC), and other cellulose ethers have been developed to be soluble in water [9,10,11]. In this context, HPC is a non-ionic cellulose which exhibits good thickening, stabilizing, and film-forming properties. Moreover, HPC films are flexible without plasticizers and non-tacky when exposed to high humidity. The combination of such features makes HPC particularly suitable for applications ranging from restoration of cultural heritage to flexible electronics and the pharmaceutical industry [12,13,14]. In fact, in addition to being used in oral pharmaceutical formulations, HPC-based hydrogels were successfully used as scaffolds in tissue engineering [15]. A recent study proved the suitability of this cellulose derivative for material extrusion 3D printing technique [16], further widening possible fields of application for such material.

Given cellulose derivatives’ extensive industrial applications, the possibility of modulating and tailoring the properties of their water solutions to obtain materials and films with specific functional purposes has been widely explored by researchers. In particular, a promising approach is to dissolve nanoparticles within the cellulose mixtures to achieve hybrid cellulose nanocomposites with superior mechanical and electrical properties [17,18,19,20]. Several nanoparticles have been tested, e.g., carbon nanotubes, cellulose nanofibers, bentonite, clays, and various studies are dedicated to nanodiamond [21,22].

Detonation nanodiamond (DND) is composed of aggregates consisting of primary nanoparticles with a crystalline diamond lattice characterized by a narrow size distribution in the range of 4–5 nm. The DND particles demonstrated a number of attractive properties, such as thermal conductivity, thermal and chemical stability, abrasiveness, hardness and low electrical conductivity [23]. As regards nanocomposite materials, studies have been devoted to the insertion of DND within polymeric matrices, such as conducting polymers, elastomers, perfluorocarbons, polysiloxanes, polyurethanes, polyimides, and rubbers, to produce new advanced hybrid multifunctional materials [24,25,26].

Although the applications of nanodiamond-based systems were at first related to diamond thermal and mechanical reinforcing and stabilizing properties, many studies subsequently proved that DND alone or in form of nanocomposite can be used to produce nano-electronic components, selective adsorbents and catalysts, abrasive tools, polishing compounds, lubricants, and materials for biology and medicine [1,10,12,27,28,29,30,31].

In this context, in a previous study [1] we demonstrated how a HPC-DND nanocomposite can be used as separator layer in a symmetric supercapacitor on paper. Briefly, the supercapacitor was realized with PEDOT:PSS electrodes, and its performance was tested as it is, i.e., without charging the system with electrolytic species, and in an electrolyte (Na_2_SO_4_) solution, and comparable results were obtained. The separator was prepared with HPC on the basis of the common and well-studied application of this cellulose derivative on paper substrates and its suitability for the blade coating technique which was used to fabricate the device [10,12,32].

In accordance with previous studies, these results suggested that the presence of DND particles within a polymeric matrix modulates the structural and functional properties of complex hybrid materials [33,34,35]. In this context, the main focus of this study is to investigate and discuss how the interplay of the DND systems with the cellulose molecules can tailor the organization of HPC chains, modifying the rheological and charge transport properties of the hybrid HPC-DND aqueous dispersions. Those hybrid dispersions were prepared at a fixed HPC amount (5%), with a DND content up to 1% w/w. An increase in DND content would lead to an unstable dispersion resulting in the sedimentation of the diamond phase due to the presence of larger DND aggregates. The results hereinafter reported are discussed and related based on the possible interactions occurring among the large variety and amount of surface functional groups onto DND surfaces, the propylic groups of the HPC molecules and water.

In this perspective, the morphological and structural features of the DND powders were investigated by means of TEM and Raman spectroscopy, while the surface chemistry was investigated by means of XPS analysis.

In addition, rheological analyses of steady and oscillatory states were performed to investigate the molecular entanglements of the HPC-DND nanocomposite in comparison of those of the HPC. Moreover, the diffusion behavior of the cellulose molecules in the presence of DND were evaluated by means of NMR analysis to derive the (equivalent) hydrodynamic radii of both the hybrid HPC-DND and reference HPC dispersions.

The effects of the possible interaction between the diamond and the cellulose phase were deeply investigated by Cyclic Voltammetry (CV) and Electrochemical Impedance Spectroscopy (EIS) techniques.

In the light of the collected results, the proposed low-cost and green approach also paves the way to extend the spectrum of deposition to printing techniques, enlarging the range of final possible applications of HPC-DND systems.

## 2. Results and Discussion

### 2.1. Materials Structural Characterizaition

#### 2.1.1. Detonation Nanodiamond (DND)

**Figure 1:**
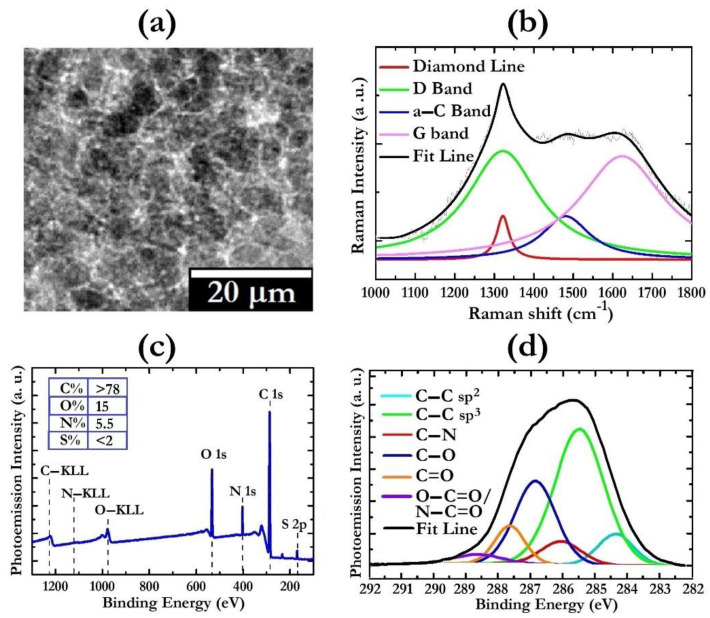
Characteristics of DND powders: (**a**) TEM image; (**b**) representative Raman spectrum obtained with laser source at 532 nm and deconvolved Raman spectrum fit with peaks’ attribution; (**c**) XPS survey spectrum showing surface elemental composition in the inset and (**d**) deconvolved high resolution XPS spectrum of C 1s core levels with signal attribution.

The morphological, structural, and surface chemistry characterizations of DND powder are reported in Figure 1.

In analogy with previous studies [29,30], the TEM image of DND powder evidences the presence of closely agglomerated grains which are sized a few hundred nanometers (Figure 1a). The formation of these aggregates is due to the strong tendency to self-assemble typical of DND particles. The Raman spectrum shows the diamond line at ~1325 cm^−1^ [29] the disorder-induced D band at ~1340 cm^−1^ [30,36], the graphite G band at ~1590 cm^−1^ [30,36], and the amorphous carbon “α-C band” at ~1510 cm^−1^ (Figure 1b) [37,38]. The downshifting and broadening of the diamond line, with respect to the Raman mode of single-crystal diamond (1332 cm^−1^), is a typical feature of nanosized diamonds [29,30,39]. The crystalline quality of the DND particles is evaluated by the calculation of the volume fraction of the diamond phase ($\Sigma_{d}$), according with the procedure reported in [40,41]. Taking into account that the ratio between the Raman cross section of amorphous and graphitic carbon with respect to diamond is 233 under the 532 nm laser source, the calculated $\Sigma_{d}$ is around 75%. This finding is consistent with the architecture of DND particles, constituted by a diamond core surrounded by a graphitic shell. Information about the surface chemistry of DND samples is achieved by XPS studies (Figure 1c). By setting the C (1s) photoelectron binding energy at 285.1 eV as a reference for calibrating all the other peaks’ positions, the core levels N 1s and O 1s are found at energies of 401.1 eV and 531.1, respectively. The quantitative analysis of the experimental data provides a surface elemental composition of about 78% of C, 5.5% of N, and 15% of O. Traces of S (<2%) were also detected as residuals of the purification process.

A more detailed investigation of the C 1s levels at a higher energy resolution was performed to quantify the possible C configurations onto the DND surfaces. As shown in Figure 1d, the deconvolution procedure revealed the XPS signals of C-Csp^2^, C-Csp^3^, C-N, C-O, C=O and C(O)NH and/or C(O)O bonding states with positions at 284.2 eV, 285.1 eV, 285.8 eV, 286.6 eV, 287.6 eV, and 288.8 eV, respectively [30,42,43,44,45]. The standard deviation of BE is ±0.1 eV. By normalizing for the relative atomic sensitive factors, the C-Csp^3^ bonds give the greatest contribution to the total spectral features with a relative abundance of ~68%, while the C-Csp^2^, C-N, C-O, C=O and C(O)NH and/or C(O)O moieties content is evaluated around 10%, 5%, 12%, 3.5% and 1.5% respectively. Since an aqueous reaction medium is used for the hybrid HPC-DND dispersion’s formation, an extent of polar groups higher than 20% can be considered a good requirement for a stable water dispersion. Moreover, the presence of these moieties onto the DND surfaces could lead to an interaction with the -OH terminations of the cellulose molecules. In addition, the presence of hydrophobic groups on the DND particles can plausibly tailor the organization of the cellulose chains. Given such considerations, the interplay of the DND systems with the cellulose and water molecules is expected to provide the formation of stable nanocomposite materials.

#### 2.1.2. Nanocomposite Characterization

##### Rheological Measurements

Rheological measurements of steady and oscillatory states were performed for both HPC and HPC-DND dispersion.

**Figure 2:**
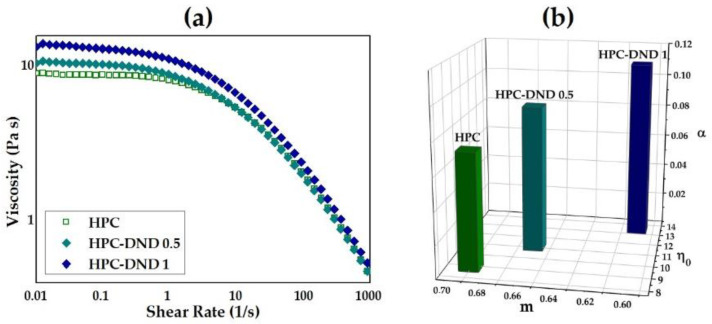
(**a**) Flow curves and (**b**) trends of the rheological parameters derived by Cross-model for HPC-DND 1, HPC-DND 0.5 and HPC samples.

As for steady state characterizations, the viscosity (*η*) of the dispersions was measured as a function of the shear rate (ɣ), in order to study its dependence on the degree of shear to which the dispersions are subjected to. The investigation on the hybrid HPC-DND and reference HPC dispersions was performed in a range of shear rates from 0.01 to 1000 s^−1^. This range of shear was chosen so as to investigate the stability of the dispersion at low shear (corresponding to the storage of the dispersions and to the leveling of the dispersion, if used as coating or thin layer) up to higher shear, compatible with the most common coating techniques [46,47,48]. Double logarithmic shear viscosity–shear rate profiles and shear stress–shear rate profiles of HPC-DND 1, HPC-DND 0.5 and HPC samples are reported in Figure 2a. The flow curves were analyzed and fitted by means of the cross-model for non-Newtonian fluids and the power law model (see Supplementary Materials (SM)) [49,50,51,52,53,54,55]. The profiles for the dispersions reported in Figure 2a are common for polymer solutions which are typically shear-thinning (pseudoplastic) fluids.

The absence of deviations in the flow curves at high shear rate (Figure 2a) indicates stability and suitability for the use of the hybrid HPC-DND dispersions. As a general trend, from Figure 2a, the viscosity decreases with the increase of the shear rates for all the investigated systems, reasonably due to the progressive uncoiling of the polymer chains bundles [56]. It is worthy to note that the viscosities of both HPC-DND 1 and HPC-DND 0.5 dispersions exceed that of the refence HPC polymer solution. As shown in Figure 2a, this behavior is especially evident at low shear rates, while for higher shear rates the differences in the viscosities values are more moderate.

To better evaluate the effects produced by the DND particles to the rheological features of the dispersions, Figure 2b shows the trend of parameters’ values (*η*_0_, α, *m*), derived by the cross-model as a function of the amount of DND. The increase of the zero-rate viscosity $\eta_{0}$ values for the nanocomposite indicates that the HPC chains’ movement at low shear are progressively impeded with the increase of DND concentration. The increase of the time constant *α* parameter’s values with the amount of DND suggests that the presence of the DND particles reduces the shear rate at which the rupture of the entanglement occurs, being the reciprocal of *α* critical shear rate of the onset of the shear thinning behavior. The index *m* decreases with the increasing of DND concentration, confirming a more evident shear thinning behavior in the presence of diamond nanoparticles. These results are in perfect agreement with the analysis of the parameters derived according to the power law (Figure S1 of Supplementary Materials).

The overall results point out that the presence of DND particles affects the HPC viscosity and the rheological properties of the dispersions. In particular, the viscosity values and the non-Newtonian fluid behavior disclosed for the hybrid HPC-DND 1 dispersions are suitable for the using of such mixtures in coating and printing processes [5,11,48,57]. In particular, the HPC-DND 1 dispersions could provide a coating runnability greater with respect to that of the HPC dispersion, due to the ‘lubricant’ effect of DND [58,59].

As expected, the greater amount of DND exerts the more significant effects, reasonably due to the fact that an increase in nanoparticles’ concentrations enhances the probability of the contact between particles and polymer. Given such considerations, the HPC dispersion containing 1% DND was chosen as the proper chemical formulation for further tests.

At this stage, additional parameters related to the structure and the strength of the dispersions are derived by oscillatory tests. Knowledge of the force or shear stress σ, the strain γ and the phase shift δ allows to completely describe the viscoelastic properties of the material. Oscillatory measurements allow for achieving information on the trend of the G′ elastic (storage) modulus and G″ viscous (loss) modulus as a function of the strain (*γ*). The ratio between the oscillatory stress (*σ*) and strain (*γ*) allows to derive the G* parameter, that measures the material overall resistance to deformation [60,61,62,63,64,65,66,67,68]. In addition, the oscillatory analysis allows to calculate the complex viscosity, η*, that contains an elastic component and a term similar to the steady state viscosity [56,61,65]. Further details and the mathematical correlation among these parameters are described in the Supplementary Materials (SM).

**Figure 3:**
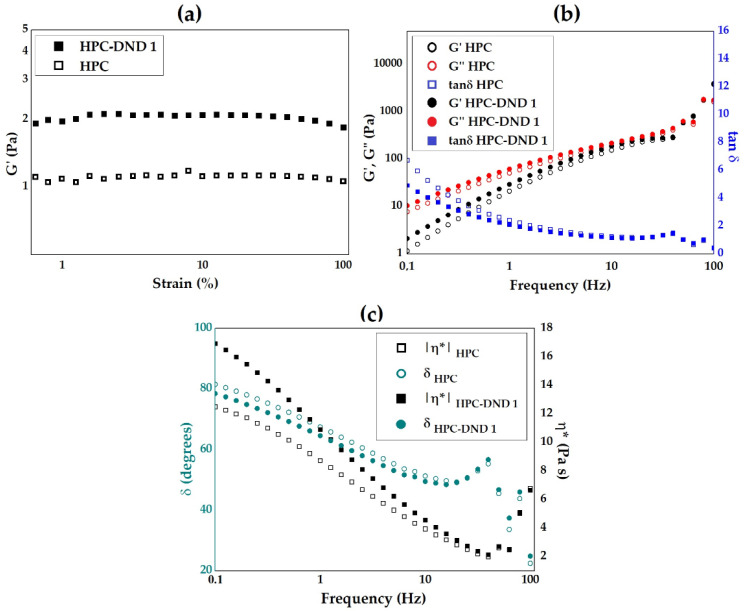
Rheological parameters of: (**a**) Storage modulus G′ vs Strain % for HPC and HPC-DND 1 samples derived from Strain sweep tests; (**b**) Storage modulus (G′), loss modulus (G″) and phase angle (tanδ) plotted against frequencies (Hz) for HPC and HPC-DND 1 samples; (**c**) Complex viscosity |η*| (Pa∙s) and δ (degrees) are reported as a function of the Frequency (Hz) for both HPC and HPC-DND 1 dispersions. HPC related data are reported as empty symbols, while HPC-DND 1 data are reported as filled symbols. Error bars are not shown for sake of clarity (errors do not affect the hierarchy of the displayed parameters).

The oscillatory characterization was performed on HPC and HPC-DND 1 samples. Preliminarily, the Linear Viscoelastic Region (LVR), which is defined by a linear relationship between the oscillatory stress (*σ*) and strain (*γ*), has been investigated through strain sweep tests, and the obtained data are reported in Figure 3a and Figure S2 of SM.

As shown in Figure 3a, the effect of the presence of the nanoparticle can be observed by the difference in the storage modulus G′ of the HPC-DND 1 with respect to that of HPC solution. In fact, the critical strain γ_c_ is at 100% and 66% strain for HPC and HPC-DND 1% respectively. This finding points out that the presence of the DND produces a strain-softening behavior of the hybrid HPC-DND 1 dispersion [66]. Frequency sweeps tested the time-dependent behavior of the samples in the non-destructive deformation range. Figure 3b report G′, G″ and tanδ values as a function of the Frequency (Hz) at a strain of 8%, which falls within the LVR previously assessed, for HPC (empty symbols) and HPC-DND 1 samples (filled symbols).

The values of the loss modulus (G″) greater than those of the storage modulus (G′) for both the HPC-DND 1 and HPC samples confirm the viscoelastic nature of these dispersions [66,67].

Considering that the G′ and G″ values in the intermediate frequency region are similar for HPC-DND 1 and HPC samples, the presence of DND particles reasonably produces no disruption effects on the entanglement network of HPC chains [61]. Correspondingly, the G′–G″ crossover point, which is where G′ = G″, is at around 50 Hz for both the samples. On the other hand, more significative changes in the shear storage modulus are found in the low frequency terminal region, which is related to the reptation relaxation which is the motion of the whole chain [60]. In this region, the slope of G′ derived for the HPC sample is steeper with respect to that of the HPC-DND 1 dispersion (Figure 3b). This finding indicates that the reptation relaxation is retarded for HPC-DND 1 dispersion, likely due to polymer–particle interactions.

In addition, the tan*δ* values slightly lower for hybrid HPC-DND 1 than for the reference HPC dispersion (Figure 3b) could be ascribed to the polymer chains being forced into more compact domains by the presence of the DND nanoparticles. Thus, the decrease in tanδ can be interpreted as a strengthening of the structure [68].

In line with the steady states’ measurements, Figure 3c shows that the complex viscosity values are higher for the hybrid HPC-DND 1 sample than for the reference HPC dispersion. As a general trend, the *δ* and η* values decrease with the increasing of the frequency for both samples. However, the decreasing of these values is steeper in the curve of HPC-DND 1 sample than that of HPC one. This trend results in the reaching of crossover points where the values are the same at around 64 and 20 Hz for *δ* and η* parameters, respectively. Analogously to the G′ and G″ modules’ analyses, the more significant differences are in the low-frequencies region, and the increase in the complex viscosity is not so dramatic as to indicate a percolative regimen.

In the view of these results, the rheological characterizations proved that the presence of the DND particles effectively modulate the viscoelastic properties of the polymer nanocomposite. In particular, the increasing of features such as shear viscosities, zero-shear viscosities, rate of shear thinning, and elastic and loss modules convincingly indicates the interaction between the diamond and cellulose phases. In this context, to qualitatively evaluate the diffusion behavior of the cellulose molecules in the presence of DND, NMR analyses were performed on the HPC and HPC-DND dispersions.

### 2.2. NMR Investigation

The interaction between two molecules in solution alters several hydrodynamic properties was analyzed. Among them, the variation in the translational diffusion behavior of a compound in the presence of another specie due to a change in size and/or shape can be used as a marker of the interaction. For this purpose, it is possible to use NMR through pulse-field gradient experiments for achieving an accurate measurement of the diffusion coefficients [69].

In the specific case of the system under study, the diffusion coefficients of HPC alone and in the presence of 0.25% and 0.5% DND were calculated. Dioxane was used as an internal standard since it is a molecule that does not interact with biopolymers such as carbohydrates or proteins [70]. In this way, it is possible to calculate an intrinsic property of the molecules under study: the hydrodynamic radius (*R_h_*) of an equivalent sphere that shows the same translational diffusion behavior. This value can be used to determine whether it changes in the presence of another specie, suggesting an interaction. On the contrary, the value of D in different conditions cannot be directly compared because it depends both on the size and shape of the molecule as well as the viscosity of the solution.

**Figure 4:**
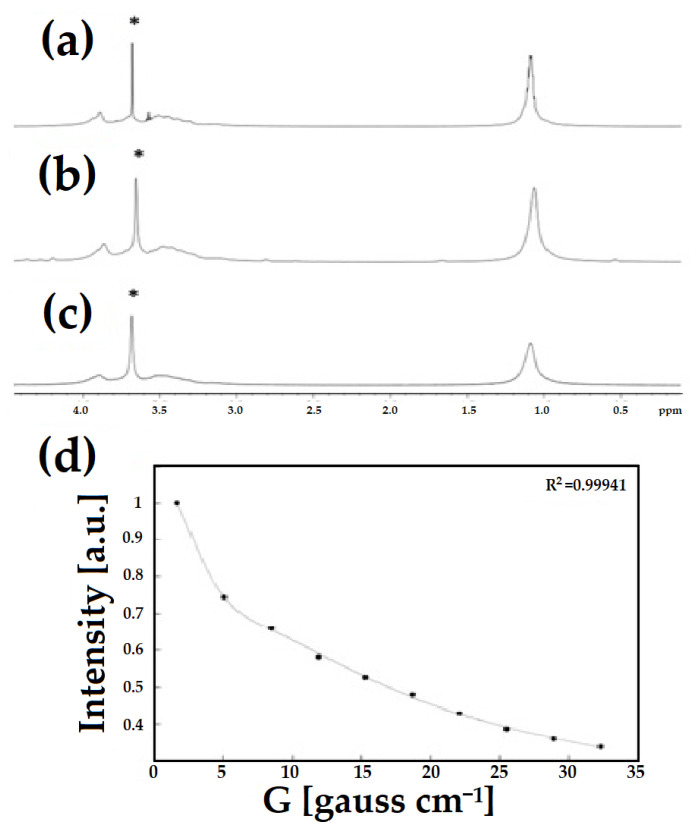
^1^H-NMR spectra obtained for solutions containing 4% of HPC and (**a**) 0%, (**b**) 0.25% and (**c**) 0.5% of DND. The peak @3.659 ppm marked with * corresponds to dioxane used as an internal standard for diffusion measurements. (**d**) Relative intensities of HPC-DND peak vs. the gradient strength applied during the diffusion experiment. The fitting was performed using a three-exponential function.

Figure 4a–c show the aliphatic part of the NMR spectra obtained for the three samples. Despite the high molecular weight of HPC, which is associated with a very short transverse relaxation time and broad signals, the significant mobility of the side chains allows for observing an intense signal at about 1.1 ppm corresponding to the methyls of the hydroxypropyl groups. The intensities of this signal and that at 3.7 ppm arising from dioxane as a function of the gradient strength were used to determine the diffusion coefficient of HPC and dioxane under the three conditions studied.

The relationship between the observed signal intensity and the power of the gradient is described by the Stejskal–Tanner equation:

(1) $$I=I_{0}exp\left[-{\left(\gamma \delta G\right)}^{2}\left(\Delta -2\delta /3\right)D\right]$$

where *I*_0_ is the signal intensity at a gradient strength of zero, *G* is the gradient strength, *D* is the diffusion coefficient, *δ* is the gradient pulse duration, and Δ is the diffusion time. As the constants *γ*, *δ*, and Δ are known, the diffusion coefficient *D* can be calculated by fitting the experimental intensities.

In the case of dioxane, this procedure allowed the diffusion coefficient to be calculated with satisfactory accuracy. Attempts to fit calculated values from Equation (1) to the observed HPC signal intensities resulted in a poor match. To obtain a reasonable agreement with the experimental data, extending the number of species present in the solution to at least three (Figure 4d) was necessary. This is not unexpected since HPC is not a homogeneous species with respect to molecular weight; therefore, this experiment yields the results of all diffusional behaviors weighted by their relative populations.

However, it is important to stress that our experimental dataset is insufficient to accurately determine all species’ relative populations and diffusion coefficients. The representation of a complex mixture of compounds like those present for *HPC* with three populations constitutes a simplified model, and our result can be considered semi-quantitative at best. Nonetheless, the fitted values showed differences between the diffusion behavior of *HPC* mixtures in the absence or presence of *DND*, which indicates an interaction.

To extract the hydrodynamic behavior of *HPC* in solution, we calculated the *R_h_* of an equivalent sphere showing the same diffusion of each species in equilibrium (Table 1), using the value of *D* calculated for dioxane and its *R_h_* as reference [71]:

(2) $$R_{h,HP\mathrm{C-}DND}=R_{h,dioxane}\frac{D_{dioxane}}{D_{HP\mathrm{C-}DND}}$$

Our approximate models indicate the presence in solution of three species with similar relative abundance with associated small (0.8–2 nm), medium (12–15 nm), and large hydrodynamic radii of the equivalent sphere. The largest species behave as a sphere with a significantly smaller hydrodynamic radius (115 nm) in the absence of *DND*; with *DND*, this radius doubles (215–238 nm). Given the r^3^ dependence on the volume, the equivalent sphere to *HPC* in the presence of *DND* shows a volume eight times larger. On the other hand, no significant differences were found between the 0.25% and 0.5% *DND* solutions, indicating that the maximum effect on *HPC* association was already obtained at the lower concentration of *DND*. This is consistent with other studies that proved that the presence of *DND* enhances the stability of cellulose-based composite materials even at concentrations lower or equal to 0.25% [12,72].

One possible way to explain this result is to consider that DND particles and/or aggregates associate with the polymer, consenting to form species with apparent larger sizes.

These results convincingly confirm that an interaction of DND particles and HPC molecules effectively occurs in an aqueous medium, providing the formation of stable aggregates within the produced hybrid HPC-DND dispersions.

### 2.3. Electrochemical and Charge Transport Properties Investigation

Although HPC is a non-ionic polymer, a small conductivity of 28 ± 2 µS cm*^−^*^1^ at 21 °C was measured for HPC 5%_w_ in water. The conductivity of DND 1%_w_ in water is 50 ± 4 µS cm*^−^*^1^ (4.0 ± 0.1 µS cm*^−^*^1^ for deionized water measured in the same condition). Despite the increased viscosity of the HPC-DND 1 sample would foresee a decreased ionic mobility, the conductivity measurements of the HPC-DND 1 provide a value of 86 ± 4 µS cm*^−^*^1^. This effect can be rationalized if we suppose a polymeric chains’ arrangement, induced by DND-polymer interaction, that facilitates the ions hopping from different groups along the chains, despite the increased viscosity of the medium [73,74]. This interesting preliminary result was further investigated by more detailed electrochemical studies.

**Figure 5:**
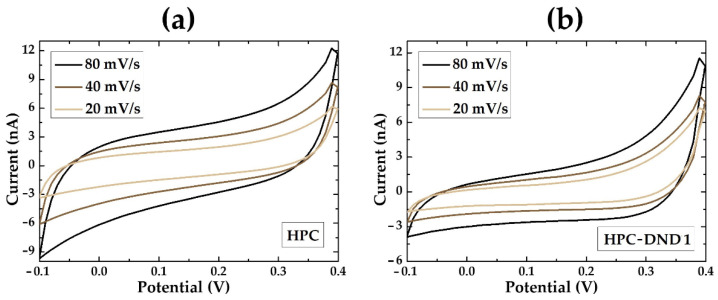
CV of (**a**) HPC and (**b**) HPC-DND 1 material at different scan rates (80 mV/s; 40 mV/s; 20 mV/s), voltage range −0.1 V to +0.4 V.

The electrochemical properties of the produced HPC-DND 1 dispersion were investigated and compared with those of reference to the HPC sample through Cyclic Voltammetry (CV) and Electrochemical Impedance Spectroscopy (EIS) analyses. Thus, the measurements are performed by placing a drop of the HPC-DND 1 or HPC dispersion on an evaporated gold electrode acquiring electrochemical responses (CV or EIS). CV voltammograms of HPC and HPC-DND 1 dispersions are shown in Figure 5.

Typical CV results for HPC and HPC-DND 1 are reported in Figure 5a,b, respectively. As clearly visible, samples with and without the addition of DND present a very similar behavior. The total absence of Faradaic processes suggests a marked stability in the explored voltage range. The difference in the curves’ area between samples with or without DND is correlated with the relationship between relative capacitances. The presence of DND significantly reduces the total capacitance of the material.

Moreover, these data suggest that the nanocomposite does not interact with the electrode in a permanent way. In fact, the measures are completely reversible, and no sign of electrode deterioration has occurred even after over 100 cycles (additional data are reported in Figure S4 of SM). This suggests that DND particles do not permanently adhere to electrode surfaces, thus allowing for the reuse of the electrode, with obvious advantages for the sustainability of the process.

**Figure 6:**
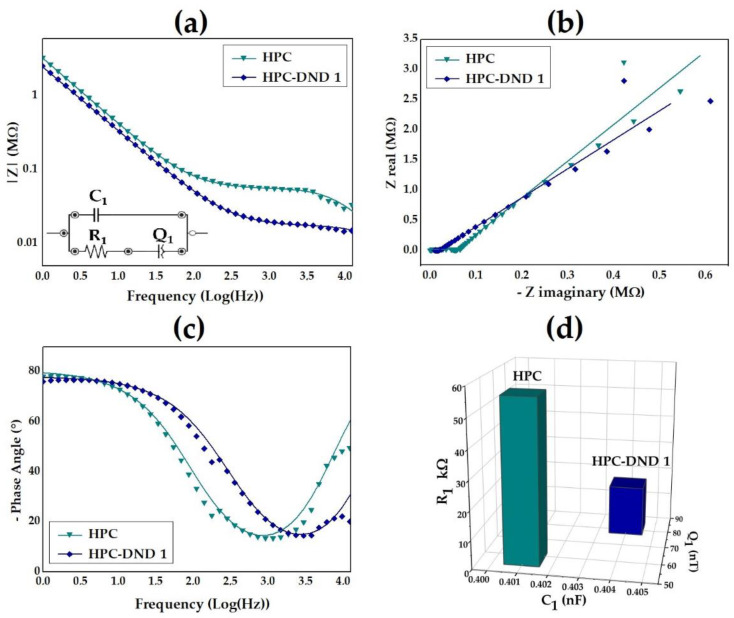
EIS experimental data (symbols) and respective fit curves (solid line) for (**a**) Bode, along with the equivalent circuit in the inset, (**b**) Nyquist and (**c**) negative phase angle plots; (**d**) trend of the EIS parameters derived from the fitting procedure for reference HPC and HPC-DND 1 samples.

The EIS experimental data are shown in Figure 6. The presence of DND in the cellulose-based material lowers the total impedance value especially in the medium- and high-frequency range (Figure 6a). This result is further confirmed by a parallel analysis conducted without the presence of cellulose, comparing water and water DND dispersion (Supplementary Materials Figure S3). The effect of the diamond is further confirmed also in the Nyquist and negative phase angle plots (Figure 6b,c). The easiest circuit able to fit the EIS experimental data is reported in the inset of Figure 6a. The latter is an adapted form of the Debye circuit which results from the parallel of an ionic resistance (R1) in series with a double layer capacitance (Q1) at the electrode surface and a dielectric capacitance (C1) [75,76].

As shown in Figure 6d, the introduction of diamond element produces no effect on the composition of the fitted circuit’s electronic components. However, the presence of DND particles within the dispersion modulates the values of the electronic elements derived from the fitting procedure, as reported in Table 2.

As shown in Table 2, the presence of DND in the dispersion lowers the total bulk resistance (R1), leaving the dielectric capacitance practically unaltered (C1). In contrast, the double layer capacitance (Q1) contribution increases as a consequence of the DND presence. These effects are in agreement with the already discussed conductivity variations and CV analyses. Therefore, the overall results suggest that the interplay between the cellulose chain and DND particles is crucial to tailor the electrical and electrochemical properties of the final material.

## 3. Conclusions

The study aims at investigating the rheological, structural, and electrochemical properties of novel water-based hydroxypropyl cellulose (HPC)-detonation nanodiamond (DND) viscous dispersions exploitable in flexible electronics. The rheological investigation disclosed that the presence of the DND affects the orientation and entanglement of cellulose chains in the aqueous medium. Features such as zero-shear viscosities, shear thinning behavior, and elastic and loss modules increase with the amount of DND. The polymeric contribution to the rheology indicates that the tested DND loadings are lower than those required for a nanoparticle percolative regimen. In line with rheological analyses, the NMR spectroscopy investigation pointed out that the presence of DND increases the equivalent hydrodynamic radius of HPC-DND dispersion with respect to that of the reference HPC sample, suggesting that the diffusion behavior of the cellulose molecules is tailored by the DND. The interplay between DND and HPC molecules likewise produces a slight increase in ionic conductivity with respect to that of the single components, despite the increased rigidity of the system, which might suggest a modification in the charge transport properties of the system. In particular, the CV evidenced that no electrochemical processes occur at the electrode, indicating a marked stability in the explored voltage range, and EIS analyses clearly evidenced that the presence of DND effectively lowers the bulk resistance of the hybrid HPC-DND material. Such a peculiarity, coupled with the possibility of producing thin layers of such material using common coating and printing techniques, results in the feasible exploitation of such systems in flexible electronic applications. Moreover, the intrinsic biocompatibility and sustainability of both HPC and DND make the produced hybrid dispersions perfect candidates for the design and fabrication of green and environmentally friendly devices.

## 4. Materials and Methods

### 4.1. Materials

Hydroxypropyl cellulose (HPC) Klucel^®^G with MW~370,000 Da was purchased from CTS. Detonation nanodiamond (DND) powders with primary sizes in the range of 5–10 nm were obtained from International Technology Center (ITC, Raleigh, NC, USA).

### 4.2. Methods

#### 4.2.1. Preparation of the Polymeric Solution and DND Composite

HPC solution was prepared by adding the proper amount of polymer (as received) to a known amount of distilled water, while mixing the solution, thus avoiding the formation of polymer aggregates. The obtained viscous solution was left for 24 h to rest.

As for the preparation of the HPC-DND composite, nanodiamonds were dispersed into deionized water. An ultrasonic bath of at least 30 min is necessary to prevent the formation of DND larger clusters. Once the DND dispersion is ready, the right amount of polymer is added, followed by prolonged manual stirring. In the following texts, polymer concentrations are quoted in % *w*/*w* values, based on the weight of dry polymer per weight of solvent (water). In order to identify the proper formulation to produce the hybrid HPC-DND nanocomposite, two DND concentrations (0.5 and 1% *w*/*w*) were tested, keeping constant the HPC amount at 5% *w*/*w*. Dispersion containing DND over 1% *w*/*w* are not investigated, due to the difficulty in obtaining a well-dispersed DND system and to the resulting sedimentation of the diamond phase in the HPC-DND dispersion, due to the presence of larger DND aggregates.

#### 4.2.2. Characterization Techniques

Rheological characterization was performed using an AR 2000 rheometer by TA Instruments. As for shear flow measurements, a cone (acrylic, 60 mm diameter 2°) and plate measurement geometry was used. The viscosity–shear rate profiles of the various dispersions studied, referred to as ‘flow curves’, were determined at 25 °C in a range of shear rates from 0.01 to 1000 s^−1^. Flow curve graphs are presented as double-logarithmic plots of viscosity (Pa∙s) vs shear rate (s^−1^). As for oscillatory measurements, a parallel plate geometry was used, with a diameter of 25 mm. Dynamic strain sweep measurements were carried out at 25 °C and 0.1 Hz, investigating a range of strain from 0.5 to 100%. Dynamic frequency sweep experiments were performed in the linear viscoelastic region (LVR) of each material with frequencies between 0.1 and 100 Hz. All experiments were repeated several times, with samples coming from different batches, and in all cases the results were reproduced within a maximum difference of 10%. Errors are not reported in the graphs hereinafter presented for the sake of clarity, and they do not affect the hierarchy of the displayed parameters.

Transmission Electron Microscopy (TEM) was employed by a Jeol 2100F apparatus operating at 200 kV and equipped with a Cs-probe corrector to determine the morphology of the samples.

Raman spectroscopy studies were performed by an Xplora ONE^TM^ (Horiba) instrument, under the following conditions: laser source at 532 nm, 10% power, 100× magnification and 3 cm^−1^ spectral resolution using a 2400T grating. The spectra are deconvolved and analyzed by means of graphical software.

An XPS analysis was performed using a Thermo Scientific K-Alpha X-ray photoelectron spectrometer with an anode using Al Kα radiation (hv = 1486.6 eV). The samples were analyzed as powder, under a basic chamber pressure of 10^−8–^10^−9^ bar. The spot size was 400 µm. The survey spectra are the average of 10 scans with a pass energy of 200.00 eV and a step size of 1 eV. The high-resolution spectra are an average of 10 scans with a pass energy of 50 eV and a step size of 0.1 eV. The XPS spectra are deconvolved and analyzed by means of Casa XPS software.

NMR experiments were performed at 298 K on a Bruker Avance 700 MHz with a 5 mm TXI probe equipped with *z*-axis gradients. Three solutions were prepared by mixing 4% HPC and increasing quantities of DND (0, 0.25%, and 0.5%). The HPC and DND concentrations were lowered with respect to 5% and 1% used for other characterization, respectively, due to the laboriousness of introducing such a viscous solution into the glass probe.

^1^H-NMR diffusion experiments were performed using a sequence double stimulated echo for convection compensation and LED using bipolar gradient pulses for diffusion in a pseudo-2D mode [77] acquiring 10 points in F1. Each spectrum was obtained after 64 scans using an acquisition time of 0.94 s and a spectral width of 15 ppm. All data were processed with TopSpin.

The conductivity of the dispersions was estimated using a Pancellet LLC GELB WEISS conductivity meter.

To perform both the CV and EIS analyses, a drop of the dispersion was placed on evaporated gold electrodes. Electrochemical analyses were carried out by means of a Palm Sense electrochemical workstation. The CVs were collected in the (−0.1 to +0.4 V) range at different scan rates (20, 40 and 80 mV/s).

The EIS measurements were performed between the frequency range of 1.0 and 11,000 Hz by applying a sinusoidal wave of 50 mV of amplitude superimposed to a 0 V bias and the resulting curves were analyzed using the proprietary software PSTrace.

All the characterizations were performed at room temperature.

## Figures and Tables

**Table 1:** Hydrodynamic radii calculated using dioxane as internal standard and *HPC* 4% at two different concentrations of *DND*. Data were fitted with a three-component exponential. The relative weights and the calculated diffusion coefficients (*D*) are also indicated.

	Molecule	Relative Population	D [m^2^s^−1^]	R_h_ [nm]
HPC 4%	dioxane		1.14 × 10^−9^	0.2 ^a^
	1	0.2	1.27 × 10^−10^	2
	2	0.4	1.60 × 10^−11^	15
	3	0.4	2.10 × 10^−12^	115
HPC 4% DND 0.25%	dioxane		1.04 × 10^−9^	0.2 ^a^
	1	0.3	2.74 × 10^−10^	0.8
	2	0.4	1.84 × 10^−11^	12
	3	0.3	9.24 × 10^−13^	238
HPC 4% DND 0.5%	dioxane		9.35 × 10^−10^	0.2 ^a^
	1	0.3	2.61 × 10^−10^	0.8
	2	0.3	1.30 × 10^−11^	15
	3	0.4	9.23 × 10^−13^	215

^a^ A fixed value of 0.212 nm was used for calculating the HPC *R_h_*.

**Table 2:** Values of electronic elements of the Debye circuits for HPC and HPC-DND 1 dispersions.

	R1	Q1	C1
HPC	56.29 kΩ	0.058 µT	0.401 nF
		0.895 Φ	
HPC-DND 1	17.57 kΩ	0.081 µT	0.404 nF
		0.869 Φ	

## Supplementary Materials

The following supporting information can be downloaded at: https://www.mdpi.com/article/10.3390/gels8120783/s1, Figure S1: Trends of the rheological parameters derived by Power law approach for the HPC-DND 0.5, HPC-DND 1 and reference HPC dispersions. Figure S2: Strain sweep results, as G′, G″ and G* vs Strain for (a) HPC; (b) for HPC-DND 1. Error bars are not shown for sake of clarity (errors do not affect the hierarchy of the displayed parameters).; Figure S3: CV curves acquired at 20, 40, 80 mV/s for (a) H_2_O and (b) DND aqueous dispersion; EIS experimental data (symbols) and respective fit curves (solid line) for (c) Bode (d) Nyquist and (e) negative phase angle plots along with the equivalent circuit in the inset; (f) trend of the EIS parameters derived from the fitting procedure for H_2_O and DND aqueous dispersion.; Figure S4: CV curves acquired at 80 mV/s for HPC-DND 1 dispersion. 110 scans were collected to confirm the reversibility of the electrode-dispersion interaction. Refs. [51,52,53,54,55,56,62,63] are cited in the Supplementary Materials.
