# Supplementary material for: A Sustainable Hydroxypropyl Cellulose-Nanodiamond Composite for Flexible Electronic Applications
Source: Gels. 2022 Nov 29;8(12):783. doi: 10.3390/gels8120783 (PMC9777684; doi:10.3390/gels8120783)
Supplement: Supplementary file 1 [file gels-08-00783-s001.zip › gels-2048420-supplementary.pdf]

gels

MDPI

Article

A Sustainable Hydroxypropyl Cellulose-Nanodiamond Composite for Flexible Electronic Applications

Elena Palmieri [1,*,†], Francesca Pescosolido [1,†], Luca Montaina [1,†], Rocco Carcione [2], Greta Petrella [1], Daniel Oscar Cicero [1], Emanuela Tamburri [1], Silvia Battistoni [2] and Silvia Orlanducci [1]

[1] Chemical Sciences Department, University of Rome "Tor Vergata", Via della Ricerca Scientifica, 00133 Rome, Italy; francesca.pescosolido@alumni.uniroma2.eu (F.P.); luca.montaina@alumni.uniroma2.eu (L.M.); petrella@scienze.uniroma2.it (G.P.); danieloscar.cicero@uniroma2.eu (D.O.C.); emanuela.tamburri@uniroma2.it (E.T.); silvia.orlanducci@uniroma2.it (S.O.)

[2] Consiglio Nazionale delle Ricerche—Institute of Materials for Electronics and Magnetism (CNR-IMEM), Parco Area delle Scienze 37A, 43124 Parma, Italy; rocco.carcione@imem.cnr.it (R.C.); silvia.battistoni@imem.cnr.it (S.B.)

* Correspondence: elena.palmieri.1@alumni.uniroma2.eu

† These authors contributed equally to this work.

Citation: Palmieri, E.; Pescosolido, F.; Montaina, L.; Carcione, R.; Petrella, G.; Cicero, D.O.; Tamburri, E.; Battistoni, S.; Orlanducci, S. A Sustainable Hydroxypropyl Cellulose-Nanodiamond Composite for Flexible Electronic Applications. *Gels* **2022**, *8*, 783. https://doi.org/10.3390/gels8120783

Academic Editor: Jean-Michel Guenet

Received: 6 November 2022
Accepted: 24 November 2022
Published: 29 November 2022

Publisher's Note: MDPI stays neutral with regard to jurisdictional claims in published maps and institutional affiliations.

Copyright: © 2022 by the authors. Licensee MDPI, Basel, Switzerland. This article is an open access article distributed under the terms and conditions of the Creative Commons Attribution (CC BY) license (https://creativecommons.org/licenses/by/4.0/).

Supplementary Materials

Rheological Investigation

To better evaluate the effects produced by the DND particles on the rheological features of the dispersions, the flow curves were analyzed and fitted by means of the Cross model, according with Equation (S1):

$$\frac{\eta - \eta_\infty}{\eta_0 - \eta_\infty} = \frac{1}{1 + (\alpha\dot{\gamma})^m} \quad (1)$$

Where η_0 is the zero-rate viscosity, η_∞ the high shear viscosity, α is a time constant associated with the rupture of linkages and m is a dimensionless parameter measuring the degree of dependence of viscosity on shear rate in the shear-thinning region. To corroborate the results achieved by the extensive Cross model, the mid-shear section of the overall flow curve was analyzed by means of the Power law (Equation (S2)):

$$\eta = k \cdot \dot{\gamma}^{n-1} \quad (2)$$

Where the constant 'k' represents the consistency of the fluid, and 'n' is a measure of how much the fluid deviates from a Newtonian fluid [1–4]. In particular, the values of the n parameter of 1, <1, or >1 are associated to Newtonian, shear-thinning, or shear-thickening behaviors, respectively. In Figure S1 the trends of the fitting parameters for hybrid HPC-DND 0.5, HPC-DND 1 and reference HPC dispersions are reported. An amplified deviation from the Newtonian behaviour (more evident shear thinning behavior) can be observed in HPC-DND samples, proved by a decrease of n values at increasing DND. Newtonian plateau is only present when the relaxation time of the deformed molecules is shorter than the time of the flow, guaranteeing the overall structure of the solution in an isotropic state. At higher shear rates, this is no longer possible because macromolecules do not have enough time to fully relax, consequently they orient along the flow direction, causing a decrease in viscosity. The increase of k values with the DND seems to indicate that the HPC chains relaxation mechanism is retarded by particles.

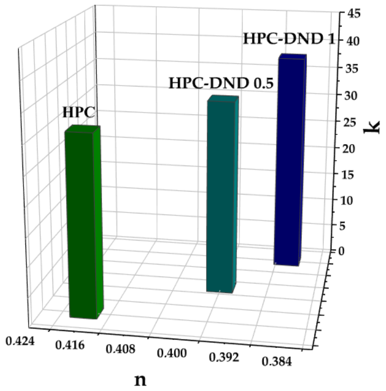

Figure S1. Trends of the rheological parameters derived by Power law approach for the HPC-DND 0.5, HPC-DND 1 and reference HPC dispersions.

Oscillatory rheology tests were performed to better understand the viscoelastic properties of the composites. Preliminarily, the Linear Viscoelastic Region (LVR), which is defined by a linear relationship between the oscillatory stress (σ) and strain (γ), has been investigated to identify the range of strain in which the material behaves independently of the strain. While small strain measurements test the sample without changing its microstructure, this could be permanently modified over a certain strain value, which is represented by the critical strain γ_c. This value can be identified by a 5% decrease of the storage modulus (G′) from the average values. After the fluid's linear viscoelastic region has been defined by a strain sweep test, its structure can be further characterized using a frequency sweep at a strain below the critical strain γ_c. This measure provides more information about the interactions among particles. For a viscoelastic material the shear force and shear strain are not in phase, but instead the deformation is delayed with respect to the force by a time quantified as the phase shift. The shift between the input stress and the output strain is the phase angle δ, which ranges from 0° to 90° for viscoelastic materials [1]. Knowledge of the shear stress σ, strain γ and the phase shift δ allows one to completely describe the viscoelastic properties of the material using G′ and G′′ parameters. G′ is the elastic (storage) modulus, that measures the elasticity of the material and its ability to store energy, in the form of elastic deformations. At strain values lower than γ_c, the elastic modulus G′ is almost independent of frequency. A higher dependency of the G′ from the frequency indicates a more fluid-like behavior of the material. On the other hand, an increased material rigidity, due to for example to increased cross-linking, causes a decrease in the slope of G′ as a function of the frequency. G′′, which is the viscous (loss) modulus, measures the ability of the materials to dissipate energy, lost as heat or as permanent deformations. G*, measures the material overall resistance to deformation and is calculated as the ratio between the oscillatory stress (σ) and strain (γ).

G′ and G′′ can be calculated from stress (σ) strain (γ) and δ according to Equation (S3)and Equation (S4), respectively.

$$G' = \frac{\vartheta}{\gamma}\cos\delta \quad (3)$$

$$G'' = \frac{\vartheta}{\gamma}\sin\delta \quad (4)$$

Tanδ measures the material damping, such as vibration or sound, and is defined as:

$$\tan\delta = \frac{G''}{G'} \quad (5)$$

For ideal solids characterized only by an elastic behavior, Tanδ = 0. In the case of real materials its value is above 1 when it is defined mainly by the viscous behavior, below 1 when the elastic response is prevailing.

The viscosity measured in an oscillatory experiment is a complex viscosity, η*, and contains an elastic component and a term similar to the steady state viscosity (Equation (S6)).

$$\eta^* = \eta' - i\eta'' \quad (6)$$

At low frequencies, η′ approaches the viscosity measured in steady shear as the shear rate approaches zero and is usually given the name 'dynamic viscosity'.

In the case of a polymer solution, elastic stresses relax sufficiently rapidly, and viscous stresses dominate, thus leading to G′′ > G′. Both parameters decrease with decreasing frequency, but the variation in G' is more abrupt. Contrarily, in the case of a gel, the stress cannot relax, showing a non-dependency of G' from the frequency. Moreover, being the gel is highly elastic, G′ > G′′ [5–8].

The Linear Viscoelastic Region was determined through strain sweep tests. In Figure S2 results from strain sweep tests are reported for both HPC 5% and HPC 5% DND 1%. In particular, the variation of G′, G′′, and G* vs Strain are reported in Figure S2 a) for HPC 5% and b) for the composite. Figure S2 shows for both the samples higher values of the loss modulus (G′′) than the storage modulus (G′), therefore they are classifiable as visco-elastic liquids, as confirmed by frequency sweep tests reported in the main text.

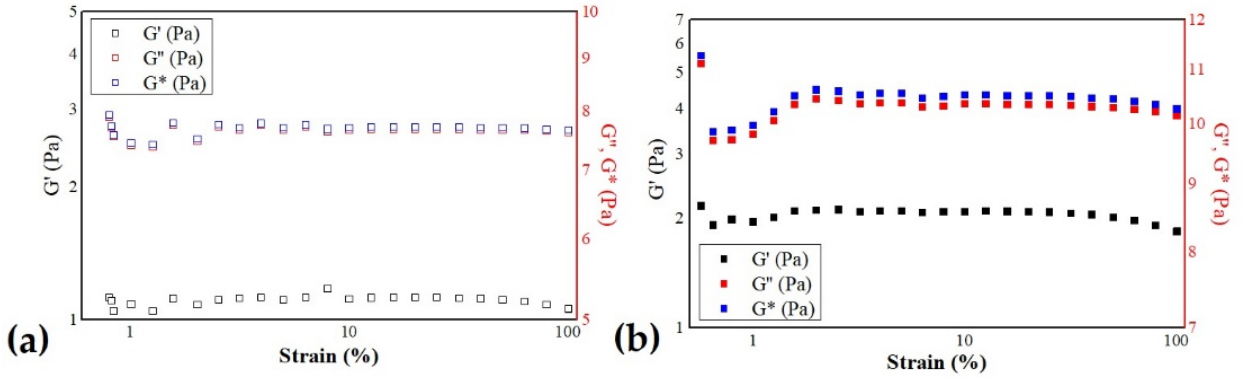

Figure S2. Strain sweep results, as G′, G′′ and G* vs Strain for (a) HPC; (b) for HPC - DND 1. Error bars are not shown for sake of clarity (errors do not affect the hierarchy of the displayed parameters).

CV and EIS results

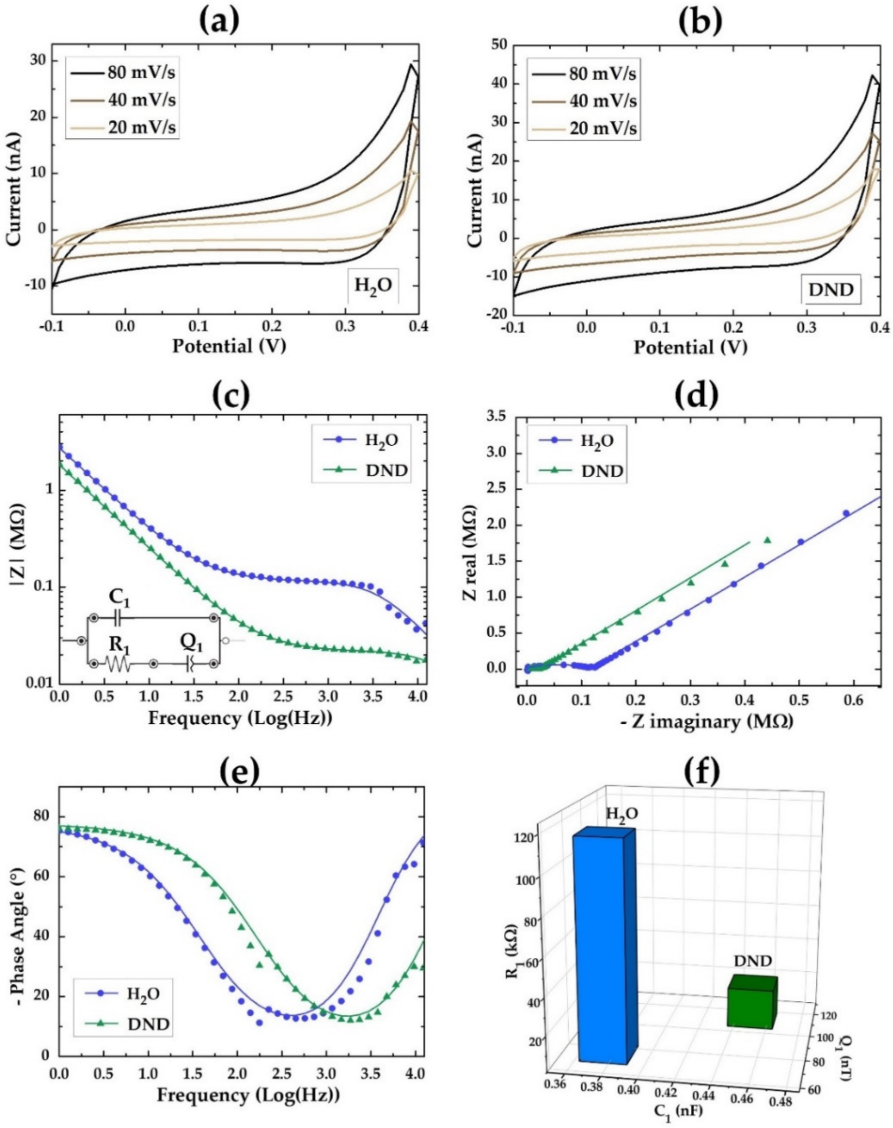

Figure S3. CV curves acquired at 20, 40, 80 mV/s for (a) H_2O and (b) DND aqueous dispersion; EIS experimental data (symbols) and respective fit curves (solid line) for (c) Bode (d) Nyquist and (e) negative phase angle plots along with the equivalent circuit in the inset; (f) trend of the EIS parameters derived from the fitting procedure for H_2O and DND aqueous dispersion.

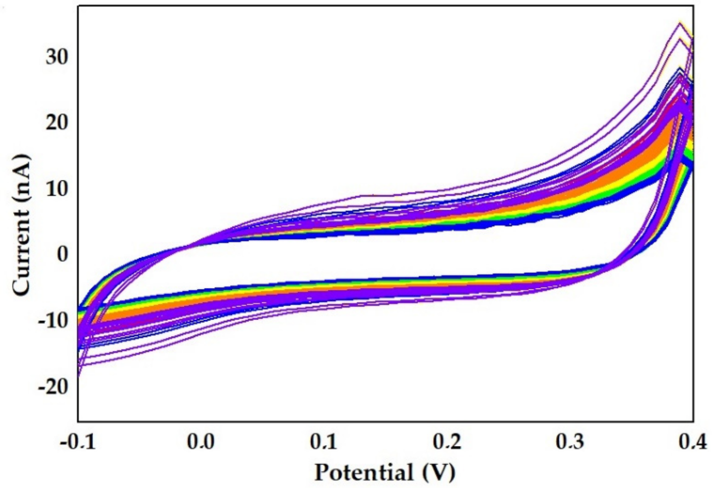

Figure S4. CV curves acquired at 80 mV/s for HPC-DND 1 dispersion. 110 scans were collected to confirm the reversibility of the electrode-dispersion interaction.

References

1. Osterhold, M. Rheological Methods for Characterising Modern Paint Systems. *Prog. Org. Coat.* **2000**, *40*, 131–137. https://doi.org/10.1016/S0300-9440(00)00124-7.
2. Marrion A. R. (Eds.), The Chemistry and Physics of Coatings, Royal Society of Chemistry, 2004. doi.org/10.1039/9781847558206.
3. Cross, M.M. Rheology of Non-Newtonian Fluids: A New Flow Equation for Pseudoplastic Systems. *J. Colloid Sci.* **1965**, *20*, 417–437. https://doi.org/10.1016/0095-8522(65)90022-X.
4. Tok, A.I.Y.; Boey, F.Y.C.; Lam, Y.C. Non-Newtonian Fluid Flow Model for Ceramic Tape Casting. *Mater. Sci. Eng. A* **2000**, *280*, 282–288. https://doi.org/10.1016/S0921-5093(99)00691-7.
5. Macosko, C. W. “Rheology. Principles, Measurements and Applications”, VCH Publishers, New York (1994). Ch. 2 pp. 65-108
6. Macosko, C. W. “Rheology. Principles, Measurements and Applications”, VCH Publishers, New York (1994). Ch. 3 pp. 109-127
7. Tschoegl, N. “The Phenomenological Theory of Linear Viscoelastic Behavior. An Introduction”, Springer-Verlag, Berlin (1989), Ch. 11, pp. 508-536
8. Lapasin, R., Pricl, S. Rheometry. In: Rheology of Industrial Polysaccharides: Theory and Applications. (1995). Springer, Boston, MA. pp. 498-578
